# Elevation of neutrophil‐derived factors in patients after multiple trauma

**DOI:** 10.1111/jcmm.17786

**Published:** 2023-06-16

**Authors:** Marie‐Therese Lingitz, Gregor Wollner, Jonas Bauer, Hannes Kuehtreiber, Michael Mildner, Dragan Copic, Daniel Bormann, Martin Direder, Claus Georg Krenn, Thomas Haider, Lukas Leopold Negrin, Hendrik jan Ankersmit

**Affiliations:** ^1^ Division of General Anesthesia and Intensive Care Medicine Department of Anesthesia Critical Care and Pain Medicine Medical University of Vienna Vienna Austria; ^2^ Department of Orthopedics and Trauma Surgery Medical University of Vienna Vienna Austria; ^3^ Department of Thoracic Surgery Medical University of Vienna Vienna Austria; ^4^ Laboratory for Cardiac and Thoracic Diagnosis and Regeneration Vienna Austria; ^5^ Department of Dermatology Medical University of Vienna Vienna Austria; ^6^ Division of Nephrology and Dialysis Department of Internal Medicine III Medical University of Vienna Vienna Austria

**Keywords:** CitH3, MPO, NE, neutrophils, trauma patients

## Abstract

Trauma represents one of the leading causes of death worldwide. Traumatic injuries elicit a dynamic inflammatory response with systemic release of inflammatory cytokines. Disbalance of this response can lead to systemic inflammatory response syndrome or compensatory anti‐inflammatory response syndrome. As neutrophils play a major role in innate immune defence and are crucial in the injury‐induced immunological response, we aimed to investigate systemic neutrophil‐derived immunomodulators in trauma patients. Therefore, serum levels of neutrophil elastase (NE), myeloperoxidase (MPO) and citrullinated histone H3 (CitH3) were quantified in patients with injury severity scores above 15. Additionally, leukocyte, platelet, fibrinogen and CRP levels were assessed. Lastly, we analysed the association of neutrophil‐derived factors with clinical severity scoring systems. Although the release of MPO, NE and CitH3 was not predictive of mortality, we found a remarkable increase in MPO and NE in trauma patients as compared with healthy controls. We also found significantly increased levels of MPO and NE on Days 1 and 5 after initial trauma in critically injured patients. Taken together, our data suggest a role for neutrophil activation in trauma. Targeting exacerbated neutrophil activation might represent a new therapeutic option for critically injured patients.

## 1 INTRODUCTION

Unintentional and violence‐related injuries are responsible for 4.4 million deaths every year, accounting for 8% of all deaths worldwide. Road traffic accidents, homicide and suicide constitute three of the top five causes of death between the ages of 5 and 29.^1^ Although Van Breugel et al.^2^ reported a decrease of approximately 1.8% per year in all‐cause mortality in polytrauma patients admitted to the intensive care unit (ICU) over the last 35 years, in‐hospital mortality remains high, at approximately 15%.^3^

After initial trauma, around 32% of patients develop multiple‐organ failure (MOF) during hospitalisation. Also, acute respiratory distress syndrome (ARDS) is one of the most common complications observed in polytraumatized patients, with reported rates of up to 50%.^3^

The endogenous response to trauma includes systemic release of pro‐ and anti‐inflammatory cytokines. Disbalance of this response can lead to systemic inflammatory response syndrome (SIRS) or compensatory anti‐inflammatory response syndrome (CARS), potentially leading to sepsis and multi organ failure.^4^, ^5^, ^6^, ^7^ While SIRS is caused by a systemic pro‐inflammatory state, CARS is a consequence of complex anti‐inflammatory signalling leading to immunosuppression. However, their underlying pathways and mechanisms remain to be completely understood,^4^, ^5^, ^8^ These distinct immunological states occur predominantly simultaneously and can lead to MOF and infections.^8^, ^9^

Neutrophils play a major role as the first line of innate immune defence and are the most abundant leukocyte in humans, constituting 60%–70% of circulating leukocytes,^8^, ^10^, ^11^ Neutrophils exert several anti‐microbial mechanisms, including phagocytosis, release of effector molecules, and formation of neutrophil extracellular traps (NETs).^12^, ^13^ NETs were first described by Brinkman et al. in 2004.^14^ Accumulations of NET‐forming activated neutrophils in damaged tissue following injury have been described.^8^, ^15^ NET formation occurs by the formation of citrullinated histones due to the activation of protein‐arginine deiminase 4 (PAD4), resulting in chromatin decondensation. Following neutrophil plasma membrane rupture, granule proteins such as myeloperoxidase (MPO) and neutrophil elastase (NE) together with intracellular DNA are released. MPO and NE additionally promote DNA decompaction.^16^ Previously, we were able to demonstrate systemic neutrophil activation after burn injury.^12^ This is in accord with other studies reporting a role for neutrophils and NETs in burn injury,^17^ critically ill patients,^11^ sepsis^18^ and lung injury.^19^ Whereas NET formation represents a protective process that captures and sequesters microbes, thereby preventing the spread of infection, a dysregulation of NET formation with increased concentration of extracellular DNA may contribute to the perpetuation of inflammation and severe tissue injury.^15^, ^20^ Although, NET formation has been reported following trauma and subsequent surgery,^21^ systemic neutrophil‐derived factors indicating NET formation such as MPO and NE have so far not been comprehensively studied in severely injured patients.

We therefore aimed to investigate systemic neutrophil‐derived immunomodulators in patients suffering from multiple injuries.

## 2 MATERIALS AND METHODS

### 2.1 Study population

In total, 106 patients were enrolled prospectively, meeting the following inclusion criteria: age over 18, admission at our urban level I trauma centre with severe injuries (injury severity score, ISS above 15) within 1 h following trauma, and primary treatment at the intensive care unit (ICU) or intermediate care unit (IMCU) with survival of at least 24 h. We excluded patients with known malignancies and chronic inflammatory lung diseases. Treatment according to the institutional standard protocol was not affected by this study. Clinical data, in‐hospital mortality and respiratory measures were recorded. For the control group, we recruited seven healthy volunteers. Patients were classified into two categories of injury severity based on the ISS (16–24 moderate, 25–74 critical) and compared to healthy controls. Data are given and plotted as median and interquartile range (IQR) given in brackets. Demographic details are presented in Table 1. The study was approved by the local ethics committee.

**TABLE 1:** Demographic details of study population.

	All patients	Survivor	Nonsurvivor	*p*‐value
*n*	106	86	19	
Age in years*	**37 [27–57]**	**34 [27–53]**	**58 [30–79]**	**0.029**
F:M ratio [%]	31:75 [29.2:70.8]	22:65 [25.3:74.7]	9:10 [47.4:52.6]	n.s.
Intubation at admittance	**58 [54.7]**	**43 [49.4]**	**15 [78.9]**	**0.023**
AIS head [%] *	3 [0–4]	2 [0–4]	5 [3–5]	**<0.001**
AIS thorax [%]	3 [2–4]	3 [2–4]	3 [3–5]	n.s.
AIS abdomen [%]	2 [0–3]	2 [0–3]	0 [0–2]	**0.041**
AIS spine [%]	1 [0–2]	0 [0–2]	0 [0–2]	n.s.
AIS extremities [%]	3 [2–4]	3 [2–3]	3 [2–4]	n.s.
AIS external [%]	1 [1–1]	1 [0–1]	1 [1–1]	n.s.
Complications [%]	41 [38.7]	30 [34.5]	11 [57.9]	n.s.
Pneumonia [%]	22 [20.8]	21 [24.1]	1 [5.3]	n.s.
ARDS [%]	8 [7.5]	5 [5.7]	3 [15.8]	n.s
*Clostridium difficile* colitis [%]	2 [1.9]	1 [1.1]	1 [5.3]	n.s.
Pancreatitis [%]	2 [1.9]	2 [2.3]	0 [0]	n.s
Urinary tract injury [%]	15 [14.2]	14 [16.1]	1 [5.3]	n.s.
Acute kidney injury [%]	13 [12.3]	9 [10.3]	4 [21.1]	n.s.
Dialysis [%]	**5 [4.7]**	**2 [2.3]**	**3 [15.8]**	**0.039**
Sepsis [%]	13 [12.3]	11 [12.6]	2 [10.5]	n.s.

*Note*: Values are the median (interquartile range) or the number (%), as indicated. Bold indicates *p* < 0.05.

Abbreviations: AIS, abbreviated injury scale; ARDS, acute respiratory distress syndrome; ISS, injury severity score.

### 2.2 Serum samples

We obtained study‐specific serum samples within the first 2 h after admission (‘day 0’, initial assessment) and then on Day 1, 3, 5, 7 and 10 after admission. Following blood withdrawal and an interval of 15–30 min to allow thorough coagulation, samples were centrifuged at 3000*g* for 15 min at room temperature. Serum samples were then aliquoted and stored at −80°C until analysed.

### 2.3 Quantification of serum NE, CitH3 and MPO

Serum NE, CitH3 and MPO were quantified by enzyme‐linked immunosorbent assay (ELISA) using commercially available kits and following the manufacturers' protocols (human neutrophil elastase, human MPO: all R&D Systems, Bio‐Techne; human CitH3, clone 11D3, Cayman Chemical). Colorimetric measurements were performed using a Tecan F50 infinite microplate reader (Tecan Group) with Magellan software (version 7.2, Tecan). Analytes were quantified according to external standard curves.

### 2.4 Statistical analysis

Statistical analyses and visualisation were performed using IBM SPSS Statistics 26.0 (IBM) and GraphPad Prism (version 9, GraphPad Software Inc.). The Mann–Whitney U‐test was used for comparison of metric and ordinal values between the two independent groups. The Kruskal–Wallis test with multiple‐comparison post hoc tests with Bonferroni correction was calculated to compare metric and ordinal values between three or more independent groups. A *p*‐value <0.05 was considered statistically significant.

## 3 RESULTS

### 3.1 Study population characteristics

The study population consisted of 31 females and 75 males, with a median age of 37 IQR: [27–57]. Nineteen patients (18%) died during hospital stay. Injured patients presented with a median ISS of 34 IQR: [24–41]. The median length of hospital stay was 34 days IQR: [17–70]. The median ICU stay was 8 days IQR: [3–21.5], with a median ventilator time of 3 days IQR: [0.75–11].

Complications during follow‐up occurred in 41 patients (38.7%). Twenty‐two patients suffered from pneumonia (20.8%) and 8 patients developed ARDS (7.5%). Urinary tract injury or acute kidney occurred in 15 patients (14.2%) and 13 patients (12.3%). Dialysis was performed in 5 patients (4.7%). Sepsis developed in 13 patients (12.3%). Further demographic and clinicopathological details are shown in Table 1. Median values of metric variables are given with interquartile range (IQR).

### 3.2 Polytrauma induces elevation of neutrophil‐derived factors

**FIGURE 1:**
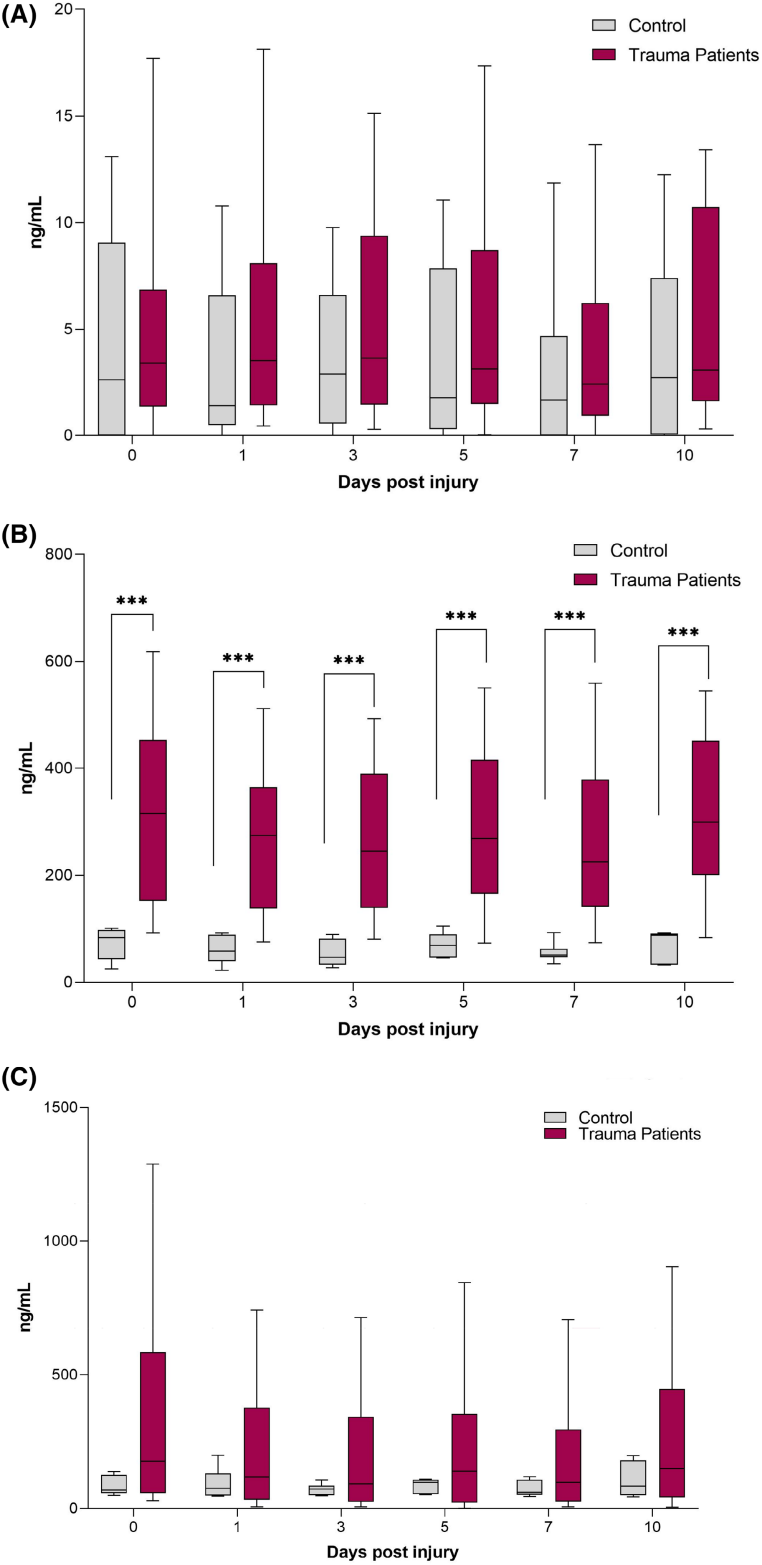
Serum levels of markers of neutrophil activation are elevated following trauma. Grouped box plots depicting the changes in serum levels of (A) CitH3, (B) MPO and (C) NE in trauma patients up to 10 days post injury. Healthy volunteers served as controls. Comparison of polytrauma patients and healthy controls, values given as median with IQR, * indicates *p* < 0.05 ** indicates *p* < 0.01, *** indicates *p* < 0.001.

Compared with healthy controls, severely injured patients demonstrated significantly elevated MPO levels from Day 0 to Day 10 after injury. (Day 0: 315.8 ng/mL, IQR: [154.6–453.2] vs. 83.4 ng/mL, IQR: [42.9–97.7], *p* = 0.001; day 1: 290.2 ng/mL, IQR: [141.5–366] vs. 58.0 ng/mL, IQR: [40.0–89.4], *p* < 0.001; day 3: 247.7 ng/mL, IQR: [142.8–391.5] vs. 46.7 ng/mL, IQR: [32.9–81.6], *p* < 0.001; day 5: 268.3 ng/mL, IQR:[166.7–411.2] vs. 69.0 ng/mL, IQR: [46.7–89.7], *p* < 0.001; day 7: 224.8 ng/mL, IQR: [148–379.2] vs. 50.6 ng/mL, IQR: [47.3–62.6], *p* < 0.001; day 10: 302.5 ng/mL, IQR: [21.8–451.9] vs. 87.8 ng/mL, IQR: [32.8–91.4], *p* < 0.001). However, no significant difference in CitH3 and NE concentrations was observed. (See Figure 1).

### 3.3 Serum concentrations of CitH3, MPO and NE in survivors versus nonsurvivors

**FIGURE 2:**
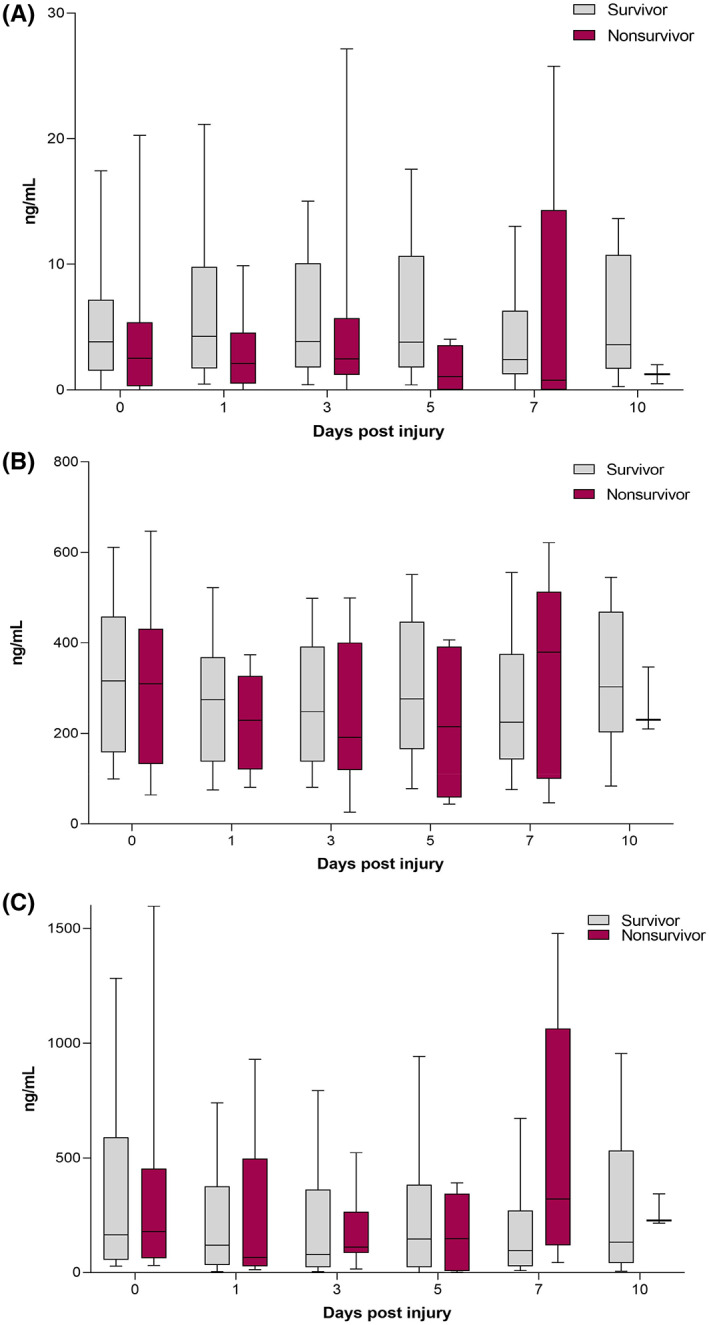
Grouped box plots depicting the changes in serum levels of (A) CitH3, (B) MPO and (C) NE in survivors and nonsurvivors up to 10 days post injury. Comparison of surviving and nonsurviving polytrauma patients; values given as median with IQR.

CitH3 levels were slightly decreased on Days 0‐10 after injury in nonsurvivors when compared to survivors. Measurements of MPO and NE showed slightly elevated serum levels of MPO and NE in nonsurvivors on Day 7 post injury (see Figure 2), but these differences were not significant.

### 3.4 Routine laboratory values in survivors versus nonsurvivors

**FIGURE 3:**
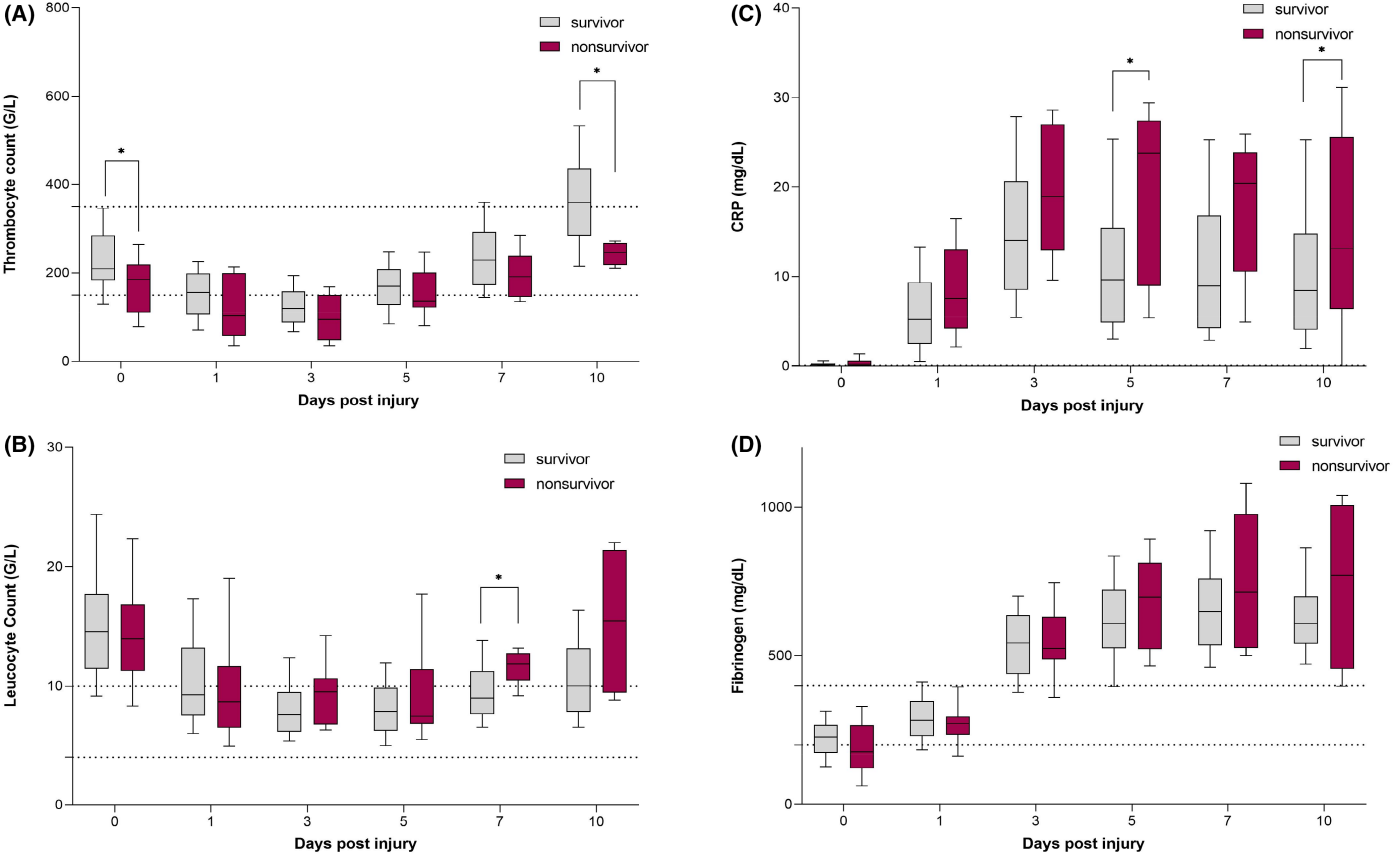
Grouped box plots depicting the changes in serum levels of (A) thrombocytes, (B) leukocytes, (C) CRP and (D) fibrinogen in survivors and up to 10 days post injury. Values given as median with IQR, * indicates *p* < 0.05.

The course of routine laboratory values from Day 0 to Day 10 after injury of survivors and nonsurvivors is displayed in Figure 3. On the day of admission (Day 0) and on Day 10 after injury, nonsurvivors of polytrauma showed significantly reduced thrombocytes (Day 0: 209 G/L, IQR: [182–285] vs. 185 G/L, IQR: [110–219], *p* = 0.036; Day 10: 361 G/L, IQR: [284–439] vs. 247 G/L, IQR: [128–268], *p* = 0.041). Additionally, nonsurvivors showed significantly elevated leukocytes on Day 7 after injury as compared with survivors (Day 7: 9 G/L, IQR: [8–11] vs. 12 G/L, IQR: [10–13], *p* = 0.033). Nonsurvivors had significantly higher serum levels of CRP on Day 5 and Day 10 post injury (Day 5: 9.63 mg/dL, IQR: [4.87–15.42] vs. 23.78 mg/dL, IQR: [8.99–27.44], *p* = 0.019; Day 10: 8.82 mg/dL, IQR: [4.51–15.41] vs 16.62 mg/dL, IQR: [12.86–28.40], *p* = 0.043). Fibrinogen:CRP ratios were also significantly reduced in nonsurvivors when compared with survivors (Table 2).

**TABLE 2:** The fibrinogen:CRP ratio was significantly reduced in nonsurvivors.

	All patients	Survivor	Nonsurvivor	*p*‐value
*n*	106	86	19	
Fibrinogen/CRP Ratio				
Day 0	**1700 [750–3721]**	**1819 [847–3906]**	**750 [467–2390]**	**0.043**
Day 1	45 [31–97]	53 [31–107]	37 [24–54]	n.s.
Day 3	32 [24–47]	33 [24–48]	24 [22–45]	n.s.
Day 5	**57 [35–93]**	**58 [41–93]**	**34 [29–64]**	**0.041**
Day 7	36 [71–120]	72 [37–128]	44 [28–83]	n.s.
Day 10	60 [42–107]	61 [43–108]	32 [31–62]	n.s.

*Note*: Values are given with median and IQR; bold indicates *p* < 0.05.

Abbreviation: CRP, C‐reactive protein.

### 3.5 NE and MPO are markedly increased in severely injured patients with ISS over 50 as compared with moderately injured patients

We found increased levels of the neutrophil‐derived factors NE and MPO in patients with higher ISS. On Days 1 and 5 after trauma, their neutrophil elastase levels were significantly increased (Day 1, moderately injured: 82.2 ng/mL, IQR: [319.0–280.7] vs. severely injured: 377.8, IQR: [241.4–810.2], *p* = 0.012; and Day 5: 133.4 ng/mL, IQR: [22.4–316.0] vs. 376.9 ng/mL, IQR: [188.9–937.0], *p* = 0.044).

**FIGURE 4:**
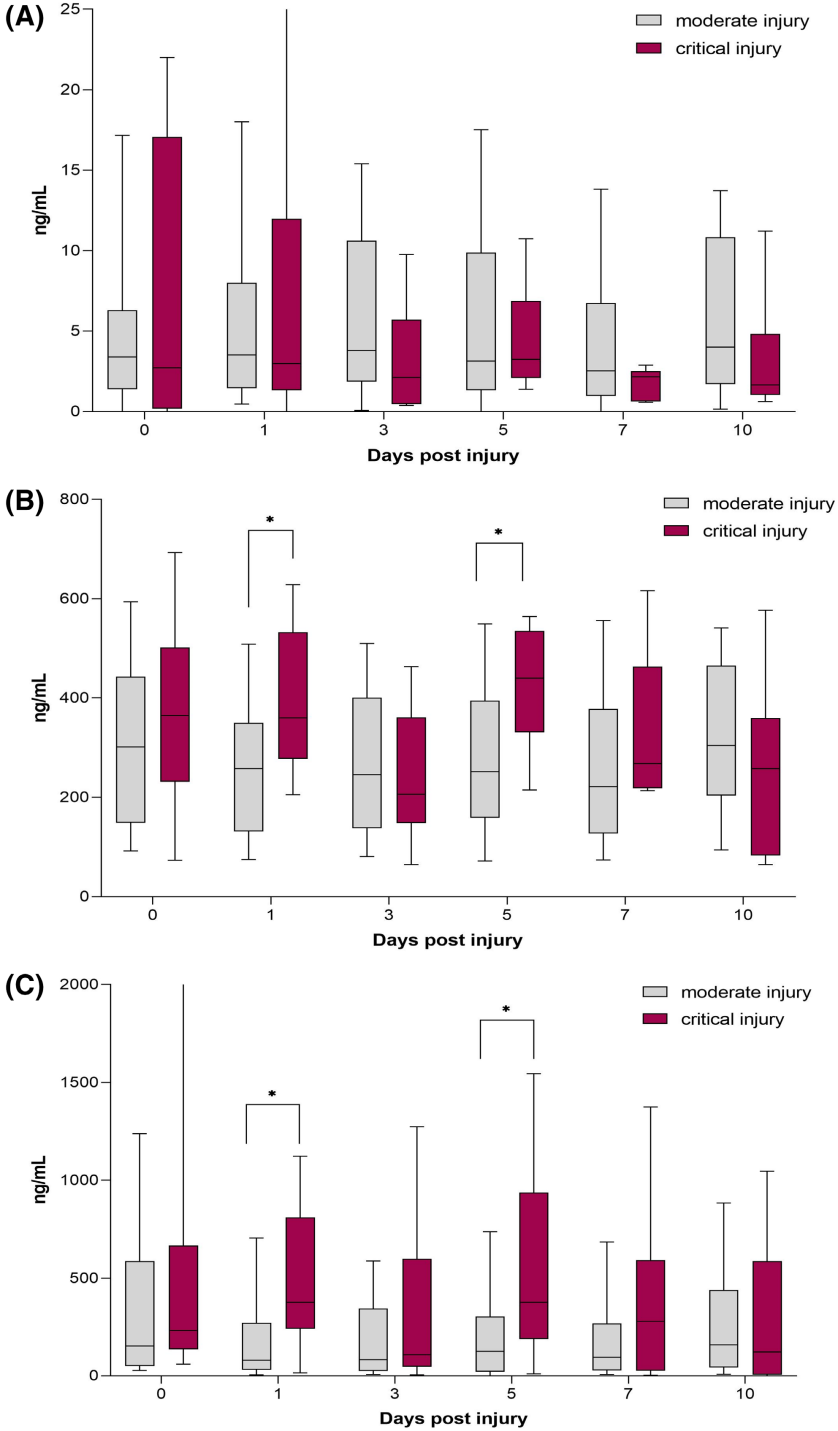
Serum levels of markers of neutrophil activation are elevated in severely injured patients. Box plots depicting the changes in serum levels of (A) CitH3, (B) MPO and (C) NE in moderately and severely injured trauma patients up to 10 days post injury. Values given as median with IQR, * indicates *p* < 0.05.

Additionally, MPO levels were significantly increased in patients with ISS over 50 on Day 1 and Day 5 after trauma (Day 1, ISS ≤50: 257.7 ng/mL, IQR: [131.1–350] vs. ISS >50: 359.6, IQR: [277.5–532.7], *p* = 0.027; and Day 5, ISS ≤50: 248.2 ng/mL, IQR: [156.6–391.8] vs. ISS >50: 440.1 ng/mL, IQR: [330.7–535.0], *p* = 0.014). (See Figure 4).

## 4 DISCUSSION

In severely injured patients, two distinct pathomechanisms with partially overlapping occurrence are known to be involved in the endogenous immunological response. The initial pro‐inflammatory state with increased secretion of pro‐inflammatory cytokines is later accompanied by an anti‐inflammatory response with increased immunological tolerance, leading to increased risk for secondary infections and late sepsis.^8^, ^9^, ^22^

Whereas many previously published studies have shown a potential role for neutrophils in patients after injury,^8^, ^15^, ^21^, ^23^ this is the first to demonstrate significantly increased serum levels of the neutrophil‐derived factor MPO in polytraumatic patients from Day 0 to Day 10 as compared with healthy controls. We also found slightly increased NE concentrations following severe trauma. Interestingly, measured serum levels of MPO, CitH3 and NE were lower than those in patients suffering from burn injuries.^12^ These results indicate a lower induction of neutrophil activation in polytraumatized patients compared with burn injury patients. Our findings are in line with the previously published study of Hirose et al.,^11^ who were able to detect CitH3‐positive cells using immunofluorescence in the bloodstream of critically ill patients. CitH3 was also identified as reliable blood biomarker for the diagnosis and treatment of endotoxic shock^24^ in a mouse model. They also indicated that CitH3 was mainly circulating in mice suffering from lipopolysaccharide shock syndrome, whereas they could rarely detect any CitH3 in mice with haemorrhagic shock. This might also serve as an explanation for the lack of a significant difference in CitH3 serum levels between severely injured patients and healthy controls.

Interestingly, CitH3 levels were reduced in surviving patients on Days 1, 5 and 10 after trauma. However, we were not able to detect significant differences in the MPO and NE serum levels of survivors versus nonsurvivors. We could only demonstrate slight increases in MPO and NE on Day 0 in nonsurviving patients, as depicted in Figure 2.

Further, we observed lower platelet counts in the early post‐injury phase, followed by a remarkable increase in platelets in the later post‐injury phase, following a U‐shaped curve. We also observed low fibrinogen levels in the early post‐injury phase, followed by a distinct increase in the later post‐injury phase. Our findings are in accord with previous observations, in which the early post‐injury phase is characterized by a hypocoagulopathic state with increased risk of bleeding and a later post‐injury phase characterized by a procoagulopathic state with increased risk for intravenous thrombosis and MOF.^25^, ^26^ Moore et al.^25^ also concluded that trauma and haemorrhagic shock are associated with a hyperfibrinolytic state, which is also in accord with our findings of initial low fibrinogen levels followed by increasing levels in the later post‐injury phase. Puranik et al.^26^ reported an incidence of coagulopathy in nearly 60% in polytrauma patients at admittance. Additionally, they demonstrated an impact of coagulation parameters such as aPTT, D‐dimer and PT on outcome in polytrauma patients. Whereas in one study fibrinogen levels under 229 mg/dL were significantly associated with increased overall mortality,^27^ in another study fibrinogen levels under 150 mg/dL measured at admission were associated with increased mortality in patients requiring massive transfusion.^28^ Although we detected no significant difference in the fibrinogen levels of survivors versus nonsurvivors, nonsurviving patients showed mean levels of 177 mg/dL (121–267) compared with 226 mg/dL (173–268) in survivors. However, we also found significantly reduced platelet counts in nonsurviving patients on Day 0 and Day 10 as compared with those in surviving patients. These findings are in line with previously published studies that reported increased mortality in patients with reduced platelet counts at admission.^29^, ^30^

We were also able to demonstrate significantly increased levels of NE on Day 1 post injury in critically injured patients (*p* = 0.046) as compared with moderately injured patients. We also found significantly decreased levels of platelets on Day 0 (*p* = 0.037) and Day 3 (*p* = 0.0007) in critically injured patients as compared with moderately injured patients. We also demonstrated highly significantly reduced fibrinogen levels on Day 0 of injury in critically injured patients when compared with moderately and severely injured patients (*p* = 0.001 and *p* = 0.008), indicating a hypocoagulopathic state with increased risk of bleeding in severely injured patients. Our findings are in accord with those of previously published work.^25^, ^26^

To our knowledge, this is the first study to describe the elevation of neutrophil‐derived factors MPO, CitH3 and NE in the post‐injury phase of polytrauma patients. It is also the first to report changes in serum levels of these investigated markers over 10 days post injury without returning to healthy controls' level. However, it should be mentioned that the results of our study are limited due to its small control group and by the fact that trauma patients suffer from numerous complications such as coagulopathy, the receipt of therapies such as extensive blood and fluid transfusion especially in critically injured patients with major bleeding events, and interventions in the course of disease, which could significantly influence neutrophil activation dynamics. We demonstrated significantly increased levels of NE on Day 1 post injury in critically injured patients when compared with moderately injured patients. Although, our study showed sustained elevations of neutrophil‐activating factors in injured patients, the exact factors that lead to neutrophil activation remain incompletely understood and further investigations are necessary to provide more direct evidence of systemic NETosis in sera of trauma patients. The results of our study may indicate that targeting exacerbated neutrophil activation might be a new therapeutic option for critically injured patients.^31^

## AUTHOR CONTRIBUTIONS

**Marie‐Therese Lingitz:** Data curation (equal); formal analysis (lead); investigation (lead); writing – original draft (lead); writing – review and editing (lead). **Gregor Wollner:** Data curation (supporting); software (supporting). **Jonas Bauer:** Data curation (equal); formal analysis (supporting); investigation (equal). **Hannes Kuehtreiber:** Data curation (supporting); investigation (supporting); writing – review and editing (supporting). **Michael Mildner:** Writing – review and editing (supporting). **Dragan Copic:** Writing – review and editing (supporting). **Daniel Bormann:** Writing – review and editing (supporting). **Martin Direder:** Writing – review and editing (supporting). **Claus Georg Krenn:** Supervision (supporting); writing – review and editing (supporting). **Thomas Haider:** Data curation (equal); supervision (lead); writing – original draft (supporting); writing – review and editing (lead). **Lukas Leopold Negrin:** Data curation (equal); project administration (lead); supervision (lead); writing – review and editing (lead). **Hendrik jan Ankersmit:** Conceptualization (lead); funding acquisition (lead); methodology (lead); resources (lead); supervision (lead); writing – review and editing (lead).

## FUNDING INFORMATION

This research project was funded by the Vienna Business Agency (Vienna, Austria; grant ‘APOSEC to clinic’ 2343727) and by the Aposcience AG under group leader H.J.A. M.M. was funded by the Sparkling Science Program of the Austrian Federal Ministry of Education, Science, and Research (SPA06/055).

## CONFLICT OF INTEREST STATEMENT

The authors declare no competing interests.

## Data Availability

Raw data are available from the corresponding authors upon request.
